# Genotype by Yield*Trait (GYT) Biplot: a Novel Approach for Genotype Selection based on Multiple Traits

**DOI:** 10.1038/s41598-018-26688-8

**Published:** 2018-05-29

**Authors:** Weikai Yan, Judith Frégeau-Reid

**Affiliations:** Ottawa Research and Development Center, Agriculture and Agri-Food Canada, 960 Carling Ave., Ottawa, Ontario, K1A 0C6 Canada

## Abstract

Genotype selection based on multiple traits is a key issue in plant breeding; it has been dependent on setting a subjective weight for each trait in index selection and a subjective truncation point for each trait in independent culling, and the weights and truncation points can be highly subjective. In this paper we proposed and demonstrated a novel approach for genotype selection based on multiple traits, the genotype by yield*trait (GYT) biplot, where “trait” can be any breeding objective other than yield; it may be an agronomic trait, a grain quality, processing quality, or nutritional quality trait, or a disease resistance. The GYT biplot ranks genotypes based on their levels in combining yield with other target traits and at the same time shows their trait profiles, i.e., their strengths and weaknesses. Compared to existing methods, this approach is graphical, objective, effective, and straightforward. Underlying the GYT biplot approach is the paradigm shift that genotypes should be evaluated by their levels in combining yield with other traits as opposed to by their levels in individual traits. An oat dataset from multi-year multi-locations trials was used to demonstrate the GYT biplot approach.

## Introduction

The importance of plant breeding to the welfare of mankind cannot be overemphasized, and genotype evaluation, i.e., identifying superior cultivars out of a population of genotypes, is a key part of this process. Genotype evaluation faces two key challenges. The first is genotype by environment interaction (GE) for a key trait, and the second is unfavorable associations among key traits^1–3^. GE has been investigated and reported in numerous publications, and a clear road map on how to handle GE in plant breeding has been outlined^4^. Briefly, data from multi-location trials in two or more years are needed to develop a strategy of dealing with GE for a given region and crop. Such multiyear multi-location data can be used to investigate whether there are any repeatable GE patterns. If yes, the patterns can be used as a guide to divide the target region into meaningful subregions or mega-environments (ME). If not, the target region should be treated as a single ME. Genotype evaluation and recommendation should be conducted for individual ME rather than across ME; thereby repeatable GE can be utilized by employing cultivars specifically adapted to each ME. By definition, GE within a ME is random noise. The noise can be canceled out and thereby genotypes be reliably evaluated if genotypes are tested in a sufficient number of trials in the ME. This number is determined by the relative size of genotypic variance versus GE variance within the ME^4^. When tested sufficiently, genotype evaluation can be based mainly on mean performance across trials and supplemented by a measure of stability. GGE (genotypic main effect plus genotype by environment interaction) biplots are an effective tool for dealing with GE for a trait^4,5^.

The current paper addresses the second challenge, i.e., genotype evaluation based on multiple traits. An ideal cultivar has to have superior levels for a number of target traits (breeding objectives). The challenge arises from the fact that target traits are usually unfavorably associated such that improvement in one trait often leads to reduced levels in one or more of other traits. Two strategies have been proposed and used in tandem or jointly, in dealing with this problem: independent culling and index selection^6–8^. Independent culling is to discard a genotype if its value for a trait is below a minimum requirement, no matter how good the genotype is for other traits. Index selection is to rank genotypes based on an index, which is a linear combination of the target traits. The difficulty with these strategies is that both are highly subjective. It is up to the breeder/researcher to set a weight for each trait in index selection and a truncation point for each trait in independent culling. The weights and truncation points can vary from researcher to researcher and from time to time for the same researcher, even for the same dataset. Different sets of weights and/or truncation points can lead to (dramatically) different selection decisions, of course.

A genotype by yield*trait (GYT) biplot approach is proposed in this paper to tackle the problem of genotype evaluation on multiple traits. It is based on the following conceptualization. 1) Yield is the most important trait and all other target traits are important only when combined with high yield. 2) The superiority of a genotype should be judged by its levels in combining yield with other target traits, rather than by its levels in individual traits. In this approach, the genotype by trait (GT) two-way table from a variety trial(s) is first transformed to a genotype by yield*trait (GYT) two-way table, in which each column is the combination of yield and a trait. The GYT table is then displayed in a GYT biplot. The average tester coordination (ATC) view^9^ of the GYT biplot is employed to rank genotypes based on their overall superiority across the yield-trait combinations and to show their trait profiles (i.e., strengths and weaknesses), which serves as the basis for genotype evaluation and recommendation.

A dataset of covered oat (*Avena sativa* L.) from Quebec, Canada will be used as an example in the case study. Covered oat is produced in Canada for human food as well as for animal feed. The hull of the covered oat grain has to be removed when used as food; the part of the oat grain after hull removal is called groat. Oat based food is regarded as healthy food as the oat groat is relatively rich in β-glucan and other soluble fibers, which have been shown to reduce the risk of heart disease, high blood pressure, and type-II diabetes when a certain amount of oat meal is served daily^10,11^. Thus, high groat percentage and β-glucan content are two important breeding objectives for milling oat, only secondary to high grain yield. In addition, good lodging resistance is a highly valued trait by oat growers; it is important for achieving high yield and good quality as well as for easy harvest. High test weight is also a valued trait by both growers and millers for easy storage and transportation. High β-glucan and low oil are desirable for use as milling oat but low β-glucan and high oil are desirable for use as feed oat. Everything being equal, high protein content, early maturity, and large kernels are also preferred. Therefore, these traits are routinely measured in oat variety trials (Table 1). It will be shown that complicated associations exist among these traits and the GYT biplot makes it easy to rank oat genotypes based on their levels of combining yield and other target traits and at the same time to show their strengths and/or weaknesses.

**Table 1:** Genotype by trait data for 26 oat cultivars for eight traits^§^.

Name	YLD (Kg ha^−1^)	GROAT (%)	BGL (%)	TW (kg hl^−1^)	LOD (0–9)^¶^	KW (g 10^−6^)	PROTEIN (%)	DTM (d)
Akina	6091	72.2	4.8	51.8	2.4	37.7	13.6	94.7
OA1426-2	6163	72.6	4.6	56.7	4.3	37.9	13.1	98.2
Nicolas	6335	73.6	4.3	53.2	3.3	36.0	13.3	95.3
Kara	6010	71.1	4.6	53.1	2.4	38.1	14.1	96.2
Noranda	5652	72.5	4.8	52.3	3.8	37.6	13.6	96.8
Unnamed1	6288	72.9	4.3	54.0	2.7	38.8	13.1	94.9
Unnamed2	5928	71.5	4.5	54.0	3.6	37.0	13.6	97.0
Nice	5836	72.3	4.5	52.7	4.4	38.5	13.3	95.1
Hidalgo	5323	73.4	4.7	52.0	5.0	33.5	13.2	94.3
Canmore	5618	70.3	4.7	54.7	4.4	39.6	14.4	94.7
Kyron	5997	70.6	4.2	52.1	2.4	36.9	13.9	95.9
Blake	5883	69.8	4.3	51.7	3.5	36.9	14.2	97.0
Kolosse	5739	74.8	4.1	53.8	2.0	37.5	13.8	95.9
OA1436-1	6201	71.9	3.9	56.6	3.8	36.0	13.3	97.4
Orrin	5589	69.9	4.4	53.4	3.8	38.2	13.4	98.1
Pomona	5870	71.6	4.0	56.4	4.2	37.3	12.5	98.2
Ruffian	5667	74.5	4.0	54.0	4.6	36.7	12.9	96.2
Oaklin	5681	71.7	4.1	53.2	4.1	38.2	13.0	94.7
Bullet	5887	71.3	4.0	55.1	3.0	39.6	13.2	96.5
Rigodon	5500	71.6	4.2	54.4	4.3	37.7	13.7	95.8
Synextra	5347	71.2	4.3	55.3	4.4	37.0	15.2	94.0
Dieter	5426	72.9	4.1	53.7	4.4	38.4	14.2	95.9
Vitality	5387	74.9	4.0	53.2	4.5	40.0	13.5	95.9
Richmond	5907	71.1	3.8	54.4	3.4	38.7	12.7	99.9
Bolina	5694	71.7	3.8	53.3	3.7	33.2	12.7	98.0
Avatar	5270	74.2	3.8	56.1	5.3	36.2	8.0	94.7
Mean	5780	72.2	4.3	53.9	3.7	37.4	13.3	96.2
Standard Deviation	302	1.4	0.3	1.5	0.9	1.6	1.2	1.5

^§^Each value is the mean across 30 trials for all traits except β-glucan content and protein content, for which each value is the mean across nine location-years. The trait abbreviations are: BGL: β-glucan content; DTM: days to maturity; GROAT: groat content; KW: kernel weight; LOD: lodging score; PROTEIN: protein content; TW: test weight; YLD: grain yield.

^¶^0 means free of lodging and 9 means lodged to flat.

## Results

### Genotype by trait (GT) biplot

**Figure 1:**
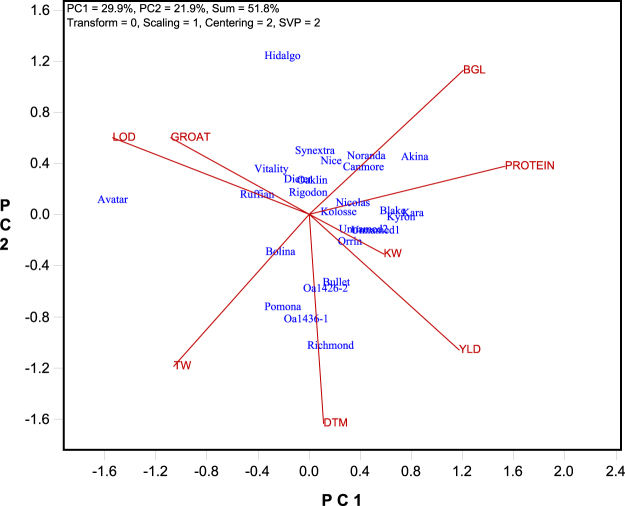
Genotype by trait (GT) biplot based on the original genotype by trait data (Table 1). The biplot was based on singular value decomposition of trait-standardized data (“Scaling = 1, Centering = 2”) and trait-focused singular value partition (“SVP = 2”). The trait codes are: BGL: β-glucan content; DTM: days to maturity; GROAT: groat content; KW: kernel weight; LOD: lodging score; PROTEIN: protein content; TW: test weight; YLD: grain yield.

The genotype by trait (GT) data presented in Table 1 are trait means for each of 26 genotypes tested across 30 trials at nine Quebec locations plus one Ontario location in 2015 to 2017. The Pearson correlations among these traits are presented in Table 2. This GT data are approximately displayed in a GT biplot^12^ (Fig. 1), which can be used to visualize the associations among traits and the trait profiles of the genotypes. The GT biplot was based on trait-standardized GT data (indicated by “Scaling = 1” and “Centering = 2” on the biplot) and trait-focused singular value partitioning (indicated by “SVP = 2”). A biplot with such settings has the following interpretations. 1) The cosine of the angle between the vectors of two traits approximates the Pearson correlation between them. Thus, an angle smaller than 90° indicates a positive correlation, an angle greater than 90° indicates a negative correlation, and an angle of 90° indicates zero correlation. 2) The angle between a genotype and a trait indicates the relative level of the genotype for the trait. Thus, an acute angle indicates that the genotype is above-average for the trait; an obtuse angle indicates that the genotype is below-average for the trait; and a right angle indicates that the genotype is average for the trait. 3) The vector length (i.e., the distance to the biplot origin) of a trait indicates how well the trait is represented in the biplot; a relatively short vector indicates that the variation of the trait across genotypes is either small or not well presented in the biplot, which is due to its weak or lack of correlation with other traits. This can occur when the goodness of fit of the biplot is relatively poor (the goodness of fit of the GT biplot in Fig. 1 is 51.8%). 4) The vector length of a genotype indicates whether it is intermediate for all traits or has clear strengths and/or weaknesses in its trait profile.

**Table 2:** Pearson correlations between traits across 26 genotypes^§^.

Traits	GROAT	BGL	TW	LOD	KW	PROTEIN	DTM
YLD	−0.18	0.13	0.03	−0.64	0.08	0.16	0.29
GROAT		−0.19	0.06	0.21	−0.12	−0.37	−0.30
BGL			−0.45	−0.13	0.07	0.42	−0.30
TW				0.32	0.11	−0.33	0.27
LOD					−0.17	−0.36	−0.14
KW						0.23	0.01
PROTEIN							−0.05

^§^The trait abbreviations are: BGL: β-glucan content; DTM: days to maturity; GROAT: groat content; KW: kernel weight; LOD: lodging score; PROTEIN: protein content; TW: test weight; YLD: grain yield. The threshold correlation for P < 0.05 is 0.396, and that for P < 0.01 is 0.502.

Based on these principles, the following observations can be made from Fig. 1. (1) Grain yield (YLD) was negatively correlated with lodging score (LOD) (a larger lodging score indicates more lodging and less lodging resistance) and groat content (GROAT) but it was not strongly associated with other traits. So good lodging resistance was important for high yielding; and grain yield and groat content was unfavorably associated. (2) Groat content was positively correlated with lodging score but negatively correlated with β-glucan content (BGL), protein content (PROTEIN), and grain yield, all being unfavorable associations. This indicates that high groat content was poorly combined with other breeding objectives in the tested cultivars. Groat content was also negatively correlated with days to maturity (DTM), meaning that earlier genotypes tended to have higher groat content. (3) β-glucan content was positively correlated with protein content but negatively correlated with test weight (TW), days to maturity, lodging score, and groat content. The negative correlations of β-glucan content with test weight and groat content are challenging unfavorable associations. (4) Kernel weight (KW) was not strongly correlated with any traits, as suggested by its short vector. These statements can be verified from the correlation table (Table 2), even though the goodness of fit of the biplot was only moderate (51.8%).

The GT biplot in Fig. 1 also shows the trait profiles of the genotypes, the accuracy of which depends on the goodness of fit of the biplot. For example, it shows that cultivar Avatar had high groat content and high test weight but low grain yield and low protein content, and it was highly susceptible to lodging; cultivar Hidalgo had high levels of β-glucan content, groat content, and lodging score and had low levels of test weight, days to maturity, and grain yield; Richmond had a trait profile quite opposite to that of Hidalgo.

Despite its usefulness in revealing associations among traits and trait profiles of genotypes, the GT biplot is not very helpful in making decisions on which cultivars to select or recommend and which cultivars to discard or discommend, which are decisions a breeder/researcher must make. The proposed GYT biplot described below was designed to accomplish this.

### Genotype by yield*trait (GYT) biplot

**Figure 2:**
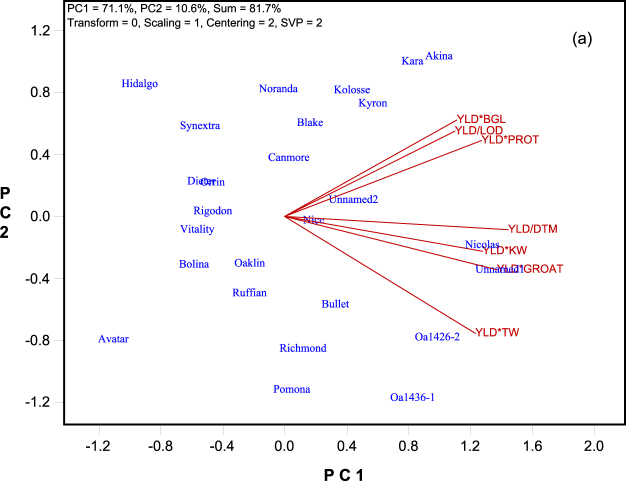
The Tester Vector view of the genotype by yield*trait (GYT) biplot to show associations among the yield-trait combinations. The biplot was based on singular value decomposition of the standardized GYT table (“Scaling = 1, Centering = 2”). The trait-focused singular value partition (“SVP = 2”) was used. The trait codes are: BGL: β-glucan content; DTM: days to maturity; GROAT: groat content; KW: kernel weight; LOD: lodging score; PROTEIN: protein content; TW: test weight; YLD: grain yield.

**Figure 3:**
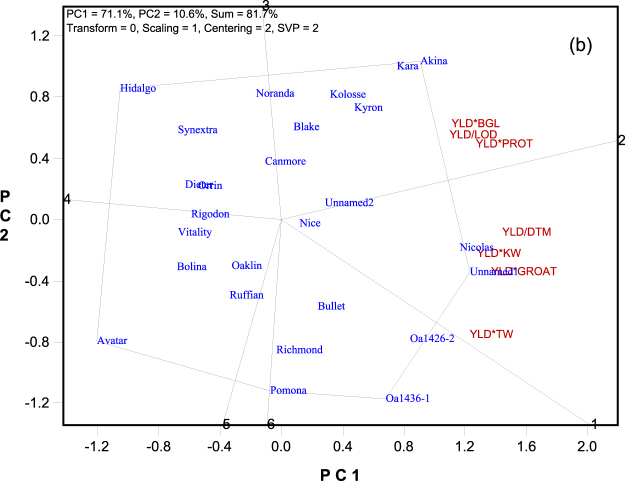
The which-won-where view of the genotype by yield*trait (GYT) biplot to highlight genotypes with outstanding profiles. The biplot was based on singular value decomposition of the standardized GYT table (“Scaling = 1, Centering = 2”). The trait-focused singular value partition (“SVP = 2”) was used. The trait codes are: BGL: β-glucan content; DTM: days to maturity; GROAT: groat content; KW: kernel weight; LOD: lodging score; PROTEIN: protein content; TW: test weight; YLD: grain yield.

**Figure 4:**
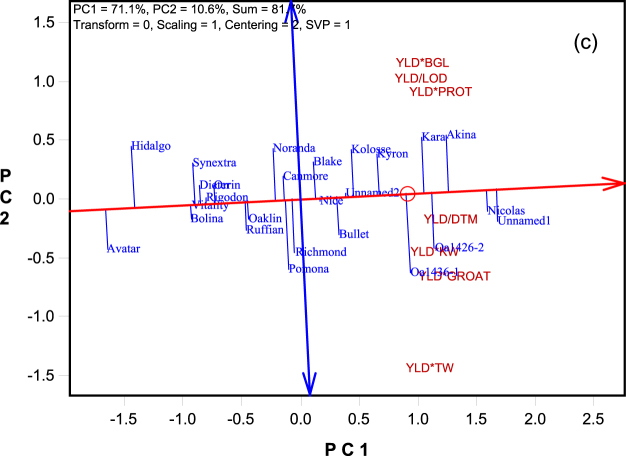
The Average Tester Coordination view of the genotype by yield*trait (GYT) biplot to rank the the genotypes based on their overall superiority and their strengths and weaknesses. The biplot was based on singular value decomposition of the standardized GYT table (“Scaling = 1, Centering = 2”). The genotype-focused singular value partition (“SVP = 1”) was used. The trait codes are: BGL: β-glucan content; DTM: days to maturity; GROAT: groat content; KW: kernel weight; LOD: lodging score; PROTEIN: protein content; TW: test weight; YLD: grain yield.

From the original GT table (Table 1), a GYT table was derived (Table 3), in which each column was a yield-trait combination. For example, YLD*BGL is the combined level of grain yield and β-glucan content, which is a measure of how grain yield and β-glucan content were combined in a genotype. Either low grain yield or low β-glucan content would affect this combined value and the genotype will thereby be judged unfavorably. The same is true for other yield-trait combinations. The combinations yield*earliness (YLD/DTM) and yield*lodging resistance (YLD/LOD) had the division operator (“/”), as opposed to the multiplication operator (“*”) in other trait combinations, to reflect the fact that more days to maturity and a larger lodging score are less desirable. The “/” operator means the values of the trait were reversed before being multiplied to the yield values. Thus, in the GYT table a larger value is always more desirable. The GYT biplot (Fig. 2) graphically displays the GYT data (Table 3), and the different views of the GYT biplot (Figs 2, 3 and 4) allows the data to be investigated from different angles. Note that yield per se was not included in the GYT data or the GYT biplot as it was incorporated into each of the yield-trait combinations.

**Table 3:** Genotype by yield*trait (GYT) data for 26 oat cultivars^§^.

Name	YLD*GROAT	YLD*BGL	YLD*TW	YLD/LOD	YLD*KW	YLD*PROT	YLD/DTM
Akina	4398	290	3155	2492	2294	598	64
OA1426-2	4473	282	3494	1450	2335	586	63
Nicolas	4666	275	3368	1931	2278	620	67
Kara	4276	279	3190	2555	2289	601	62
Noranda	4100	272	2955	1505	2126	557	58
Unnamed1	4586	269	3393	2288	2441	601	66
Unnamed2	4237	264	3200	1657	2191	576	61
Nice	4217	261	3078	1319	2247	561	61
Hidalgo	3909	251	2767	1069	1784	514	56
Canmore	3951	262	3073	1286	2222	571	59
Kyron	4233	254	3124	2499	2214	588	63
Blake	4107	256	3041	1697	2174	584	61
Kolosse	4290	234	3088	2817	2151	593	60
OA1436-1	4455	241	3508	1645	2234	594	64
Orrin	3909	244	2985	1485	2135	525	57
Pomona	4201	237	3309	1389	2187	527	60
Ruffian	4224	228	3059	1243	2078	544	59
Oaklin	4071	234	3023	1382	2168	530	60
Bullet	4200	234	3243	1989	2330	552	61
Rigodon	3938	232	2994	1279	2073	540	57
Synextra	3809	228	2957	1219	1977	579	57
Dieter	3954	222	2914	1246	2085	561	57
Vitality	4037	216	2868	1195	2152	543	56
Richmond	4200	226	3216	1747	2283	534	59
Bolina	4085	218	3037	1530	1890	518	58
Avatar	3911	201	2956	990	1907	486	56
Mean	4171	247	3115	1650	2163	561	60
Standard Deviation	220	23	187	504	149	33	3

^§^The trait abbreviations are: BGL: β-glucan content; DTM: days to maturity; GROAT: groat content; KW: kernel weight; LOD: lodging score; PROTEIN: protein content; TW: test weight; YLD: grain yield. The units for the yield-trait combinations are not important as it is the standardized data that is used in genotype evaluation.

### Associations among various yield-trait combinations

Since all yield-trait combinations have yield as a component, they tend to be positively correlated with each other, as indicated by the acute angles in the biplot (Fig. 2). This is an important feature of the GYT biplot, as opposed to the GT biplot (Fig. 1); it allowed genotypes to be graphically and meaningfully ranked based on their yield-trait combinations (below). Nevertheless, strong trait associations observed in the GT biplot (Fig. 1), e.g., the positive correlation between β-glucan content and protein content and the negative correlations of test weight with these two traits (Fig. 1 and Table 2) can still be seen in the GYT biplot, as shown by the magnitudes of angles among YLD*TW, YLD*PROT, and YLD*BGL.

### Trait profiles of the genotypes

Figure 3 is the polygon view or “which-won-where” view^9^ of the same biplot as in Fig. 2. This view is particularly useful for visualizing the trait profiles of the genotypes. The irregular polygon was formed by connecting the genotypes with the longest vectors in all directions. For each polygon side a line was drawn to start from the biplot origin and to be perpendicular to the polygon side. These lines divided the yield-trait combinations into two sectors; corresponding to each sector there was a polygon vertex. The geometry of the biplot determines that the genotype placed on a vertex has the largest values for the yield-trait combinations placed within the corresponding sector. Thus, Akina (and closely placed Kara) had the largest values for YLD*BGL, YLD*PROT, and YLD/LOD, meaning that these two cultivars were the best in combining grain yield with β-glucan content, protein content, and lodging resistance. Similarly, Unnamed1 (and closely placed Nicolas) had the highest levels of YLD/DTM, YLD*KW, YLD*GROAT, and YLD*TW, meaning that these two cultivars were the best in combining grain yield with early maturity, kernel weight, groat content, and test weight. From Fig. 3 it is also apparent that OA1436-1 had a contrasting trait profile to that of Akina and Kara although all three cultivars had good levels of yield.

### Superiority rank of the genotypes based on their yield-trait combinations

Figure 4 is the ATC view of the same biplot as Figs 2 and 3 except that it was based on genotype-focused singular value partitioning (indicated by “SVP = 1” on the biplot), so as to focus on comparison among genotypes^13^. The small circle in the biplot represents the placement of the “average yield-trait combination,” which is determined by the coordinates of all yield-trait combinations included in the biplot. The line with a single arrow passes through the biplot origin and the average yield-trait combination and is called the average tester axis (ATA). The arrow points to higher mean values for the genotypes, across all yield-trait combinations. The ATA serves the purpose of ranking the genotypes based on their overall superiority or usefulness. The line with two arrows pointing outwards passes through the biplot origin and is perpendicular to the ATA. This double-arrowed line serves to separate genotypes better than average (placed on its right, on the same side as the ATA arrow) from those poorer than average (placed on the left side). This separation intuitively suggests the researcher to focus on the genotypes ranked better than average. The double-arrowed line also helps indicate whether a genotype had an all-rounded or balanced trait profile or had obvious strengths and/or weaknesses; the latter determines how a “useful” genotype should be used in terms of environmental adaptation and/or end use. Genotypes placed close to ATA (i.e., with short projections to the double-arrowed line) tend to have balanced trait profiles whereas those placed away from the ATA in either direction tend to have obvious strengths and/or weaknesses.

From Fig. 4, the best ranked cultivars based on the yield-trait combinations included: Unnamed1 > Nicolas > Akina > OA1426-2 > Kara > OA1436-1. Avatar and Hidalgo, placed on the far left side of the biplot, were ranked the poorest, even though they were among the best in groat content (Table 1). In addition to ranking genotypes based on their overall superiority, Fig. 4 also shows the trait profiles of the genotypes (although Fig. 3 is the best for this purpose). Specifically, Fig. 4 shows that Nicolas and Unnamed1 were balanced for various traits; Akina and Kara were strong in β-glucan content, protein content, and lodging resistance but poor in test weight; and OA1436-1 was strong in test weight but poor in β-glucan content, protein content, and lodging resistance. This information is important for deploying the superior but different cultivars to their most suitable environments and end uses. In addition, regardless of their overall superiority, all genotypes placed below the ATA tended to have relatively good levels of test weight, groat content, kernel weight, and/or early maturity, but relatively low levels of β-glucan, lodging resistance, and/or protein content. The opposite is true for genotypes placed above the ATA.

### Cultivar evaluation based on the GGE biplot for yield vs. that on the GYT biplot for multiple traits

**Figure 5:**
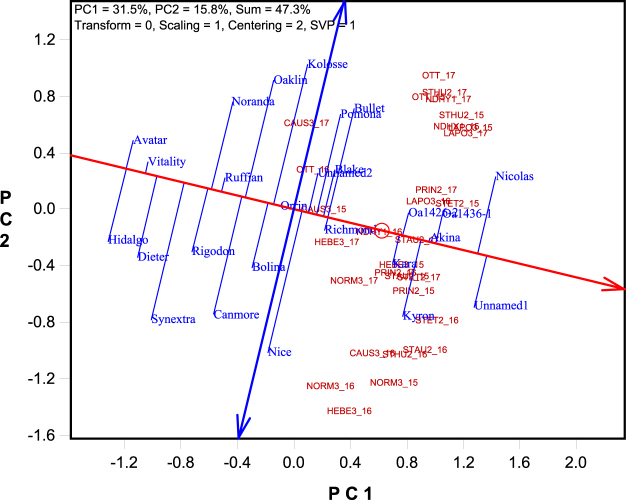
Genotypic main effect plus genotype by environment interaction (GGE) biplot of grain yield for 26 genotypes in 30 environments. The biplot was based on environment standardized data and genotype-focused singular value partition (“Scaling = 1”, “Centering = 2”, and “SVP = 1”). Each environment is represented by a location code jointed with a year code. For example, OTT_17 represents the trial at Ottawa in 2017. The locations codes are: CAUS3: Causapscal; HEBE3: Hébertville; LAPO3: La Pocatière; NDHY1: Notre-Dame de Saint-Hyacinthe; NORM3: Normandin; OTT: Ottawa (Ontario), PINT2: Pintendre; PRIN2: Princeville; STAU2: Saint-Augustin;; STET: St. Etienne; STHU: St Huber; STRO1: Sainte-Rosalie; STS1: Saint-Simon.

Presented in Fig. 5 is the ATC view of the GGE biplot for grain yield for the 26 cultivars tested in the 30 trials. No repeatable GE patterns can be seen in the GGE biplot, meaning that the 30 trials should be regarded as random samples of a single ME. The ATC view of the GGE biplot is therefore suitable for evaluating the genotypes on their mean yield and stability across the environments. The ATA points to higher mean yield and the double-arrowed line points to greater instability in either direction. Seven cultivars showed clear yield advantage over other cultivars. They were: Unnamed1 > Nicolas > OA1436-1 > Akina > Kyron > OA1426-2 > Kara. It can be noted that this rank is different from that based on the GYT biplot (Fig. 4). Among the seven high yielding cultivars, Kyron and OA1436-1 were ranked lower in the GYT biplot, due to their poor levels in combining yield with groat content, β-glucan content, and/or test weight. The rank change between the GGE biplot for yield and the GYT biplot for multiple traits highlighted and validated the usefulness of the GYT biplot in identifying superior cultivars; superior cultivars must be high yielding but not all high yielding cultivars are superior for a given end use.

## Discussion

Although numerous papers have been published and continue to be published on GE analysis of single traits, publications on genotype evaluation based on multiple traits are few. This may be interpreted as that genotype evaluation based on multiple traits is no longer an issue. As senior plant breeders the authors can testify that this is not the case. The fact is that plant breeders and statisticians working with them have chosen to accept the reality that this issue is too complicated to tackle and there are no better ways other than depending on the breeder/researcher’s personal judgement to set a subjective weight and a subjective truncation point for each trait when making selection decisions. The GYT biplot proposed in this paper provides a novel approach to genotype evaluation based on multiple traits. This approach is comprehensive and effective, as it graphically ranks the genotypes based on their levels in combining yield with various target traits and at the same time shows the strengths and weaknesses of the genotypes. The rank indicates the usefulness of the genotypes and the strengths and weaknesses suggest how the genotypes should be used. This approach is objective because no subjective weights and truncation points are involved. The selection results depend only on the traits that are included in the analysis. It is advisable to include only those traits that are essential for the success of a cultivar in GYT biplot analysis.

One novelty of this approach is the paradigm shift that the superiority of a genotype should not only be measured by its levels in individual traits but more importantly by its levels in combining yield with other target traits. This paradigm shift emphasizes the importance of yield relative to other breeding targets, which is in line with the common sense and practice in plant breeding and cultivar evaluation. Indeed, yield is the only trait that can determine the usefulness of a genotype by itself while other traits (agronomic traits, quality traits, or disease resistances) are valuable to producers only when they are combined with sufficiently good yield levels. For example, an oat genotype with a β-glucan level of 8% would be a highly valuable breeding parent. However, if its yield is only 50% of the best cultivars, then it will not be an acceptable cultivar. Similarly, a genotype with extremely good lodging resistance but very low yield would have no place in growers’ fields. The same can be said of all other traits. Thus, levels of yield-trait combinations are more meaningful than levels in individual traits in selecting superior cultivars (though not necessarily so in selecting breeding parents). The relation between yield and other target traits for a crop cultivar may be compared to that between the skin and the hair for a fur; a trait gains its value only when associated with a yield level.

Another novelty of the proposed approach is its use of the ATC graph of the biplot in multi-trait analysis. The ATC view was initially developed for GGE biplots such that genotypes can be visually evaluated for their mean performance and stability across environments for a trait^9^. However, this view is valid only when the following conditions are met: 1) the data from all environments (or columns in the two-way table, in a generic term) have the same unit (or unit-free in case of standardized data), and 2) there are no strong negative correlations between individual environments and the average environment. For a GT biplot (Fig. 1), the first condition is met because it is based on trait-standardized data, but the second condition is rarely met due to strong negative correlations among traits. Also, in the GT data (Table 1) some traits are so presented that a large value means less desirable, which makes the ATC view meaningless. However, these conditions are all met in the GYT biplot (Fig. 2), making the ATC view of the GYT biplot a meaningful and effective tool to rank genotypes based on various yield-trait combinations and to show the strengths and weaknesses of the genotypes.

The GYT biplot analysis is straightforward because the yield-trait combinations can be readily calculated from the GT data and because biplot analysis is now routinely used by many researchers. For those who are not yet using biplot analysis, a superiority index integrating all yield-trait combinations can be easily calculated using a spreadsheet. This involves a few simple steps: 1) generating the GYT table (Table 3) from the GT table (Table 1), 2) standardizing the GYT table to form a standardized GYT table (Table 4), and finally, 3) taking the mean across the standardized yield-trait combination values for each genotype, which can be used to rank the genotypes (last column, Table 4). The strengths and weaknesses of each genotype can be appreciated by examining Table 4 as well. In fact, the GYT biplot (Fig. 2) is simply a graphical approximation of the standardized GYT data (Table 4). Nevertheless, the GYT biplot is highly recommended as it is much more effective than the GYT table.

**Table 4:** Standardized genotype by yield*trait (GYT) data and superiority index for the genotypes^§^.

Cultivars	YLD*GROAT	YLD*BGL	YLD*TW	YLD/LOD	YLD*KW	YLD*PROT	YLD/DTM	Mean (Superiority Index)
Unnamed1	1.89	0.96	1.48	1.27	1.86	1.22	2.03	1.53
Nicolas	2.25	1.22	1.35	0.56	0.77	1.78	2.12	1.43
Akina	1.03	1.88	0.21	1.67	0.88	1.11	1.39	1.17
Oa1426-2	1.37	1.52	2.03	−0.40	1.15	0.77	0.88	1.05
Kara	0.48	1.41	0.40	1.80	0.84	1.20	0.78	0.99
Oa1436-1	1.29	−0.26	2.10	−0.01	0.48	1.00	1.18	0.83
Kyron	0.28	0.33	0.05	1.69	0.34	0.80	0.82	0.62
Kolosse	0.54	−0.53	−0.14	2.32	−0.08	0.96	−0.07	0.43
Unnamed2	0.30	0.76	0.45	0.01	0.18	0.46	0.34	0.36
Bullet	0.13	−0.56	0.68	0.67	1.11	−0.26	0.31	0.30
Nice	0.21	0.62	−0.20	−0.66	0.56	0.01	0.42	0.14
Blake	−0.29	0.39	−0.40	0.09	0.07	0.68	0.19	0.11
Richmond	0.13	−0.88	0.54	0.19	0.81	−0.81	−0.32	−0.05
Pomona	0.14	−0.40	1.04	−0.52	0.16	−1.03	−0.11	−0.10
Canmore	−1.00	0.66	−0.23	−0.72	0.39	0.30	−0.26	−0.12
Noranda	−0.32	1.12	−0.85	−0.29	−0.25	−0.13	−0.56	−0.18
Oaklin	−0.45	−0.56	−0.49	−0.53	0.03	−0.92	−0.03	−0.42
Ruffian	0.24	−0.81	−0.30	−0.81	−0.57	−0.50	−0.38	−0.45
Orrin	−1.19	−0.09	−0.69	−0.33	−0.19	−1.08	−1.03	−0.66
Rigodon	−1.06	−0.61	−0.65	−0.74	−0.61	−0.63	−0.87	−0.74
Dieter	−0.99	−1.04	−1.07	−0.80	−0.52	0.02	−1.16	−0.79
Synextra	−1.64	−0.80	−0.85	−0.86	−1.25	0.55	−1.06	−0.84
Vitality	−0.61	−1.31	−1.32	−0.90	−0.07	−0.53	−1.30	−0.86
Bolina	−0.39	−1.24	−0.42	−0.24	−1.83	−1.30	−0.65	−0.87
Hidalgo	−1.19	0.20	−1.86	−1.15	−2.54	−1.41	−1.20	−1.31
Avatar	−1.18	−1.96	−0.85	−1.31	−1.72	−2.25	−1.46	−1.53
Mean	0	0.0	0.0	0.0	0.0	0.0	0.0	
Standard Deviation	1	1.0	1.0	1.0	1.0	1.0	1.0	

^§^The trait abbreviations are: BGL: β-glucan content; DTM: days to maturity; GROAT: groat content; KW: kernel weight; LOD: lodging score; PROTEIN: protein content; TW: test weight; YLD: grain yield.

It may be argued that GYT approach puts too much weight on yield relative to other traits. However, this approach reflects the consideration and reality of the oat value chain (and possibly the value chains of other crops). The first consideration of oat growers in choosing oat cultivars is their yield levels, as soon as they meet the minimum quality requirements from the end users. Although millers benefit directly from high quality (high groat content and high β-glucan content, in particular), they also understand the importance of grain yield to oat growers such that high grain yield combined with best possible quality is also their criterion when recommending oat cultivars. Their purpose of doing so is to ensure a reliable supply of oat grain with sufficiently good quality at regular prices, as opposed to a supply of best quality grain at higher prices. Moreover, the GYT biplot does allow the choices of oat cultivars for specific adaptations and end uses. For example, Fig. 4 shows that Nicolas and Unnamed1 ranked the best and had all-rounded or balanced trait profiles, and therefore can be recommended as all-purpose cultivars in Quebec and similar regions. Akina and Kara were good in combining yield with β-glucan, protein, and lodging resistance, though poor in test weight. They are therefore more suitable for use as milling oat for environments where lodging is a key problem. In contrast, OA1436-1 was good in combining yield with test weight, but was poor in β-glucan, protein, and lodging resistance. It is therefore more suitable for use as feed and for growing in environments where lodging is less of a problem.

## Methods

### The data source

The sample dataset (Table 1) was derived from the 2015 to 2017 Quebec provincial oat registration and recommendation trials, organized by Réseaux Grandes Cultures du Québec (RGCQ) and Centre de recherche sur les grains inc. (CÉROM). These trials were conducted annually at nine locations representing the crop zones of Quebec, plus at Ottawa, Ontario, making up 10 locations each year. A randomized complete block design with three replications was used in each trial. Each year about 45 covered oat cultivars or breeding lines were tested, and 26 cultivars were tested in all three years. In addition to grain yield, data on agronomic traits (days to maturity, plant height, lodging score) and grain quality traits (kernel weight, test weight, and hull percentage, which is the reverse of groat content) were collected for each genotype at all locations. Groat content, β-glucan content, oil content, and protein content were determined for composite samples across replications for each genotype from three locations each year. The data in Table 1 are mean values for each genotype-trait combination across the trials.

### The genotype by yield*trait (GYT) table

The GYT table (Table 3) was obtained as follows. For groat content, β-glucan content, protein content, test weight, and kernel weight, the values for the yield-trait combinations were obtained by multiplying the yield value with the trait value for each genotype (e.g., YLD*BGL). For lodging score and days to maturity, which were so measured that a larger value means less desirable, the values for the yield-trait combinations were obtained by dividing the yield value with the trait value for each genotype (e.g., YLD/LOD). Some traits, e.g., lodging and disease scores, are usually measured with 0 as the best and a larger value is less desirable. In this case it is advisable to reverse the values such that 0 means worst and a larger value means more desirable before calculating the yield*trait values. This ensures that in the GYT table a larger value is always more desirable. The units for the yield-trait combinations are not important as it is the standardized data that are used in genotype evaluation.

### Data standardization

The GT table or the GYT table was standardized so that the mean for each trait or yield-trait combination becomes 0 and the variance becomes unit (e.g., see Table 4). The standardization was performed as:

1 $${P}_{ij}=\frac{{T}_{ij}-{\bar{T}}_{j}}{{s}_{j}},$$

where *P*_*ij*_ is the standardized value of genotype *i* for trait or yield-trait combination *j* in the standardized table, *T*_*ij*_ is the original value of genotype *i* for trait or yield-trait combination *j* in the GT or GYT table (Tables 1 and 3), ${\bar{T}}_{j}$ is the mean across genotypes for trait or yield-trait combination *j*, and *s*_*j*_ is the standard deviation for trait or yield-trait combination *j*.

### Construction of a GT biplot

The GT biplot (Fig. 1) was based on the first two principal components (PC) resulting from singular value decomposition (SVD) of the standardized GT table. SVD decomposes the GT table into genotype eigenvalues, trait eigenvalues, and singular values:

2 $${{\rm{P}}}_{{\rm{ij}}}=({d{\rm{\lambda }}}_{1}^{{\rm{\alpha }}}{{\rm{\zeta }}}_{{\rm{i}}1})({{\rm{\lambda }}}_{1}^{1-{\rm{\alpha }}}{{\rm{\tau }}}_{1{\rm{j}}}/{\rm{d}})+({d{\rm{\lambda }}}_{2}^{{\rm{\alpha }}}{{\rm{\zeta }}}_{{\rm{i}}2})({{\rm{\lambda }}}_{2}^{1-{\rm{\alpha }}}{{\rm{\tau }}}_{2{\rm{j}}}/{\rm{d}})+{{\rm{\varepsilon }}}_{{\rm{ij}}}$$

where *ζ*_*i1*_ and *ζ*_*i2*_ are the eigenvalues for PC1 and PC2, respectively, for genotype *i*; *τ*_*1j*_ and *τ*_*2j*_ are the eigenvalues for PC1 and PC2, respectively for trait *j*, and *ε*_*ij*_ is the residual from fitting the PC1 and PC2 for genotype *i* on trait *j*; λ_1_ and λ_2_ are the singular values for PC1 and PC2, respectively. α is the singular value partitioning factor. When α = 1 (i.e., SVP = 1 in terms of GGEbiplot), the biplot is said to be genotype-focused, and is suitable for comparing genotypes. When α = 0 (i.e., SVP = 2), the biplot is said to be trait-focused, and is suitable for visualizing correlations among traits. Genotype by trait relations are not affected by the choice of α. The scalar d is chosen such that the length of the longest vector among genotypes is equal to that that among traits, which is important for generating a functional biplot^3^. The GT biplot was constructed by plotting $({d{\rm{\lambda }}}_{1}^{{\rm{\alpha }}}{{\rm{\zeta }}}_{{\rm{i}}1})$ against $({d{\rm{\lambda }}}_{2}^{{\rm{\alpha }}}{{\rm{\zeta }}}_{{\rm{i}}2})$ for genotypes and plotting $({{\rm{\lambda }}}_{1}^{1-{\rm{\alpha }}}{{\rm{\tau }}}_{1{\rm{j}}}/{\rm{d}})$ against $({{\rm{\lambda }}}_{2}^{1-{\rm{\alpha }}}{{\rm{\tau }}}_{2{\rm{j}}}/{\rm{d}})$ for traits in the same plot.

### Construction of a GYT biplot

The procedures for constructing a GYT biplot (Fig. 2) are exactly the same as constructing a GT biplot except the term “trait” should be replaced with “yield-trait combination.”

### Construction of a GGE biplot

The GGE biplot (Fig. 5) presented in this paper was generated the same way as the GT biplot (Fig. 1) except that the term “trait” is replaced with “environment.” It is useful to note that there are different types of GGE biplots, depending on how the data are scaled before being subjected to SVD^3^. The GT biplot, GYT biplot, and GGE biplot were generated using the GGEbiplot software^3^. A recent addition to this software is to directly transform a GT biplot into a GYT biplot.

### Data availability statement

All relevant data are included in the manuscript.

### Ethical approval and informed consent

This work has no bearing on ethical issues.
